# Open-source platforms to investigate analytical flexibility in neuroimaging

**DOI:** 10.1162/IMAG.a.79

**Published:** 2025-07-21

**Authors:** Jacob Sanz-Robinson, Michelle Wang, Brent McPherson, Yohan Chatelain, David Kennedy, Tristan Glatard, Jean-Baptiste Poline

**Affiliations:** NeuroDataScience-ORIGAMI Lab, McConnell Brain Imaging Centre, The Neuro (Montreal Neurological Institute-Hospital), Faculty of Medicine and Health Sciences, McGill University, Montreal, Quebec, Canada; Department of Computer Science and Software Engineering, Concordia University, Montreal, Quebec, Canada; Department of Psychiatry, University of Massachusetts Medical School, Worcester, MA, United States; Krembil Centre for Neuroinformatics, Centre for Addiction and Mental Health, Toronto, Ontario, Canada

**Keywords:** analytical flexibility, open source, software, reproducibility, neuroimaging

## Abstract

Researchers in brain imaging have access to a multitude of analysis tools, many of which carry out the same or similar tasks but yield different results when applied to the same data. This analytical flexibility often undermines reproducibility and raises concerns about the robustness of neuroimaging studies. However, the array of software packages to investigate and address analytical flexibility is decentralized, scattered, and not well documented. Consequently, researchers often lack the necessary information and protocols to buttress the reliability of their findings across analytical tools. This review catalogs and describes software platforms (i.e., software or computational libraries) that can be used to address result variability arising from computational pipelines and environments and explores the use of computing platforms and neuroimaging pipeline frameworks in addressing this issue. This study offers guidance to the research community on accessing, understanding, and utilizing these platforms to address brain imaging analytical flexibility. Additionally, the article provides specific recommendations tailored to different user groups, considering the tools they intend to use with these platforms and their computational constraints.

## 1 Introduction

Neuroimaging aims to quantitatively investigate the structure and function of the brain and the central nervous system, but faces growing concerns regarding the reproducibility of existing studies (Bowring et al., 2019; Eklund et al., 2016; Kennedy et al., 2019; Poldrack et al., 2017). An important factor contributing to the concerns of neuroimaging result reproducibility is analytical flexibility: the range of outcomes that can be obtained across different widely used analysis methods with similar goals. Neuroimaging researchers are often faced with multiple choices of software pipelines, versions, and parameters to carry out a given computation, which may yield varying results from the same data, impacting result robustness (Bhagwat et al., 2021; Botvinik-Nezer et al., 2020; Bowring et al., 2019; Carp, 2012). Even using the same pipelines and data, result reproducibility can be impacted by the operating system (OS) used (Glatard et al., 2015), the OS version, hardware, and parallelization parameters. The propagation of numerical instabilities within pipelines can be amplified by changes to a dependent library (Kiar et al., 2020; Salari et al., 2020). Additionally, when the same pipeline is launched on data acquired from different populations or scanning sites, population drift—the change in a population’s characteristics over time due to random variations or sampling effects—can compromise result replicability (Dockès et al., 2021; Fortin et al., 2018; Kennedy et al., 2019).

Analytical flexibility challenges computational researchers trying to reach a consensus with their peers when investigating similar inquiries. Setting aside supervised learning, many studies lack a ground truth to compare against. It is generally hard to reproduce neuroimaging findings reliably across laboratories.

In computational fields, “reproducibility” often encompasses closely-related terms used throughout this review (Schloss, 2018). “Replicability” tests a result’s reliability with the same methods and code as the original study but different data. “Robustness” tests reliability with different code but the same data. “Generalizability” checks whether results hold with entirely different code and data. “Reproducibility” verifies results using the original code and data.

For example, in Botvinik-Nezer et al. (2020), 70 independent teams were provided with the same neuroimaging data to test 9 different hypotheses. Across the different teams—none of which chose identical workflows—five out of the nine hypotheses had no clear consensus with regard to significant findings. The most commonly used functional MRI analysis packages yield different results (Bowring et al., 2019). Similarly, in another study (Schilling et al., 2021), 42 independent teams were provided with the same whole-brain streamline data to segment. The teams’ differing segmentation protocols resulted in white matter pathway reconstructions that varied more across different protocols for the same subject than they did across the different subjects. When different methods produce conflicting results, it is hard to determine the most reliable approach (Bhagwat et al., 2021).

Analytical variability can also be harnessed to improve replicability, robustness, and generalizability in neuroscience research (Kiar et al., 2024). Strategies such as multiverse analyses and perturbation models are proposed to systematically investigate variability across tools, configurations, and datasets. By quantifying and managing this variability, researchers can build more robust findings and enhance scientific progress.

**Fig. 1.**
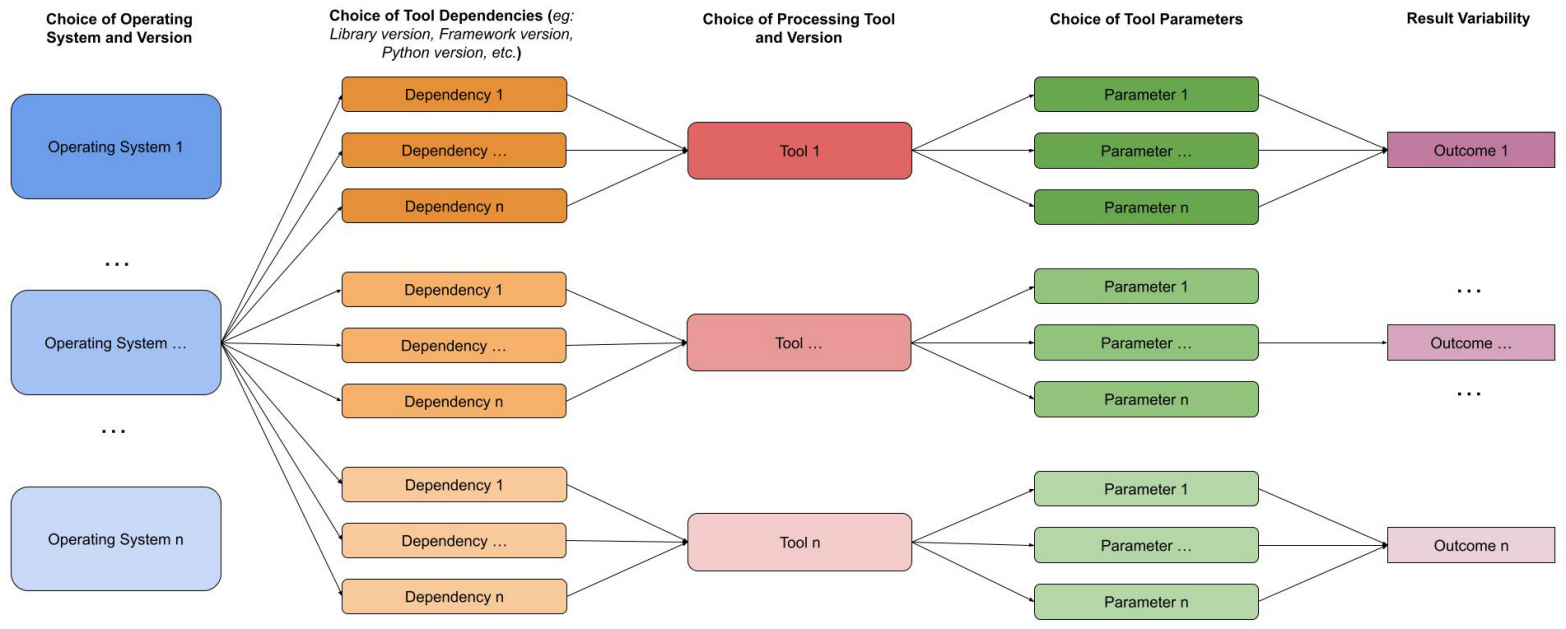
Analytical flexibility in neuroimaging workflows. Key decision points, from the operating system to dependencies, tools, and parameters, contribute to variability in outcomes, emphasizing the risks of analytical flexibility and the need for reproducibility platforms.

As neuroimaging methods progressed, platforms/software were created to help laboratories with less computational experience organize data and run pipelines. These platforms can often be leveraged to facilitate investigating the effects of analytical flexibility (Fig. 1). Platforms were also developed to assess the stability of numerical operations in response to changes in the computational environments where pipelines are executed, such as variations in the underlying OS, compiler, and libraries. However, many researchers in different teams remain unaware of these platforms and their potential benefits for improving the reproducibility of their findings.

The challenge of reproducible research extends beyond concerns about analytical flexibility. The academic publishing focus on human-readable descriptions is not conducive to adequately and efficiently verifying scientific claims in computational research. Although many data provenance platforms now exist to track a dataset’s evolution from raw sources to results in machine-readable formats—enabling reproducible analyses (Donoho, 2010; Halchenko et al., 2021; Kennedy et al., 2019) and easing the investigation of analytical variations—they are not widely used. It has been emphasized that computational reproducibility across widely used methods, such as independent component analysis (ICA) and computational morphometry, is critically impacted by variability in numerical precision, initialization parameters, and software implementations (Adali & Calhoun, 2022). These efforts collectively highlight the importance of tools that can systematically address analytical and environmental variability. As a result, assessing the robustness of results with new data or pipelines remains challenging.

Recent efforts to address reproducibility in neuroimaging have provided valuable tools and frameworks to mitigate variability in computational workflows and environments. At the article level, reporting checklists promotes transparency by standardizing methodological documentation, enabling researchers to better assess and replicate findings (Ekhtiari et al., 2024). At the analysis level, this article describes platforms that can be used to assess analytical flexibility—see for instance Adali & Calhoun (2022).

Despite growing concerns about reproducibility levels due to analytical flexibility in neuroimaging (Botvinik-Nezer & Wager, 2023), *no comprehensive resource exists to guide researchers in choosing software platforms to assess the impact of analytical variability on their results*. Rapid development and evolution of computational infrastructures make capturing available resources challenging. This review uniquely consolidates and categorizes open neuroinformatics platforms, creating a practical reference for neuroimaging researchers to assess and mitigate variability in their results. We have included all open platforms we are aware of at the time of writing, aiming to raise awareness of neuroinformatics software packages that foster reproducibility practices, and to reduce the barriers to their access, understanding, and adoption. We distinguish “analytical platforms” enabling the study of analytical flexibility and “neuroimaging tools or software” that perform analytical tasks (e.g., FSL brain segmentation, SPM co-registration).

**Fig. 2.**
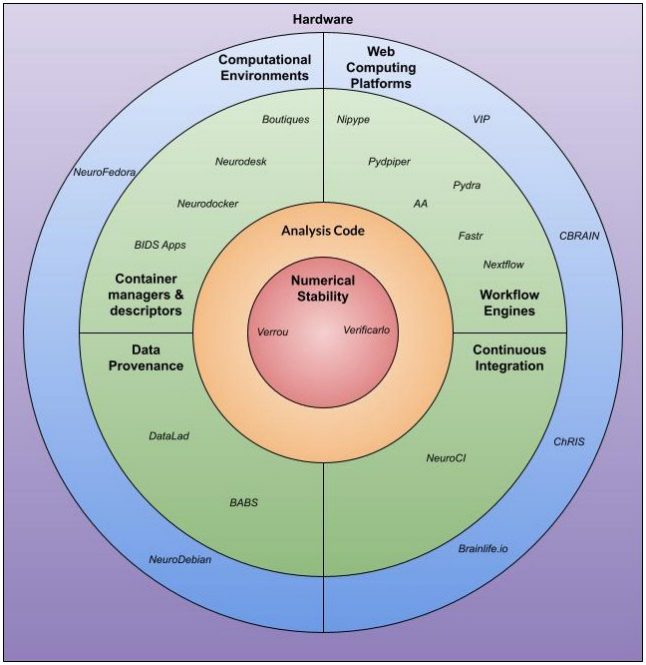
The landscape of the categories and tools featured in this review.

The platforms discussed in this review have been classified based on their capacity to assist neuroimaging researchers in exploring and addressing variability arising from two significant categories: (1) the use of different neuroimaging pipelines and parameters and (2) the use of different computational environments. We introduce the outline of this article in Table 1. The categories of platforms are shown in Figure 2, and the platforms are listed alongside links to their resources, utilized documentation for our experiments, and support groups in Table 2.

**Table 1.** The outline of the sections featured in this review.

Section	Name	Summary
1	**INTRODUCTION**	N.A.
2	**Platform Solutions for Analytical (tool) Variability in Neuroimaging Processing**	**2.1: Pipeline Containerization and Tool Repositories** **2.1.1: Container-based Solutions—**Simplify tool sharing and execution. **2.1.2: Container Descriptors—**Enable transparent and automated container usage. **2.1.3: Integrated Computational Environments—**Feature dedicated neuroimaging operating systems with preinstalled neuroimaging tools.
		**2.2: Workflow Engines** Facilitate the organization and re-execution of analytical computation sequences.
		**2.3: Continuous Integration** Automate computations across pipelines and datasets.
3	**Data Provenance**	Software that enables the transparent logging of dataset transformations.
4	**Numerical Stability**	Software to investigate the stability of mathematical floating-point operations with regard to environmental variations.
5	**Web Computing Platforms**	Browser-accessible platforms that provide instant access to tools via graphical interfaces, bypassing traditional programming requirements for remote analyses.

**Table 2.** Links to the platforms to investigate analytical flexibility in neuroimaging, their documentation, and corresponding support groups.

Section	PlatformName	Platform Link	Utilized Documentation for Experiments	Support Group Link (Slack, Mattermost, forum, mailing list, etc.)
2.1.1	BIDS Apps	https://bids-apps.neuroimaging.io	https://github.com/bids-apps/antsCorticalThickness https://github.com/bids-apps/freesurfer	https://mattermost.brainhack.org/brainhack/channels/bids_general https://groups.google.com/g/bids-discussion
	Neurodocker	https://www.repronim.org/neurodocker	https://www.repronim.org/neurodocker/user_guide/examples.html	https://github.com/ReproNim/neurodocker/discussions
	Neurodesk	https://www.neurodesk.org	https://www.neurodesk.org/docs/	https://github.com/orgs/NeuroDesk/discussions
2.1.2	Boutiques	https://boutiques.github.io	https://boutiques.github.io/doc/ https://nbviewer.org/github/boutiques/tutorial/blob/master/notebooks/boutiques-tutorial.ipynb	https://github.com/boutiques/boutiques/issues
2.1.3	NeuroDebian	https://neuro.debian.net	Same as “Platform Link”	https://github.com/neurodebian/neurodebian/issues
	NeuroFedora	https://docs.fedoraproject.org/en-US/neurofedora/overview	https://docs.fedoraproject.org/en-US/neurofedora/install-media/	Multiple channels listed at https://docs.fedoraproject.org/en-US/neurofedora/communicating/
2.2	Nipype	https://nipype.readthedocs.io	https://nipype.readthedocs.io/en/latest/quickstart.html# https://miykael.github.io/nipype_tutorial/notebooks/introduction_quickstart.html	https://brainhack.slack.com/messages/C1FR76RAL https://app.gitter.im/#/room/#nipy_nipype:gitter.im
	Pydpiper	https://github.com/Mouse-Imaging-Centre/pydpiper	https://wiki.mouseimaging.ca/display/MICePub/Pydpiper https://github.com/Mouse-Imaging-Centre/pydpiper/blob/main/INSTALL	https://github.com/Mouse-Imaging-Centre/pydpiper/issues
	AA	https://github.com/automaticanalysis/automaticanalysis	https://github.com/bids-apps/aa	https://github.com/automaticanalysis/automaticanalysis/discussions
	Fastr	https://fastr.readthedocs.io	https://fastr.readthedocs.io/en/stable/static/quick_start.html	https://groups.google.com/g/fastr-users
	Nextflow	https://www.nextflow.io	https://training.nextflow.io/latest/basic_training/ https://www.nextflow.io/docs/latest/index.html	https://www.nextflow.io/slack-invite.html
	Pydra	https://pydra.readthedocs.io	https://github.com/nipype/pydra-tutorial/tree/master/notebooks	https://github.com/nipype/pydra/discussions
2.3	NeuroCI	https://github.com/neurodatascience/NeuroCI	https://github.com/neurodatascience/NeuroCI/tree/old-version-cbrain	https://github.com/neurodatascience/NeuroCI/issues
3	DataLad	https://www.datalad.org	https://docs.datalad.org/ https://handbook.datalad.org/	https://matrix.to/#/%23datalad:matrix.org
	BABS	https://pennlinc-babs.readthedocs.io	https://pennlinc-babs.readthedocs.io/en/stable/walkthrough.html	https://github.com/PennLINC/babs/discussions
4	Verrou	https://github.com/edf-hpc/verrou	Same as “Platform Link”	https://github.com/edf-hpc/verrou/issues
	Verificarlo	https://github.com/verificarlo/verificarlo	https://github.com/verificarlo/fuzzy	https://github.com/verificarlo/verificarlo/discussions
5	CBRAIN	https://cbrain.ca	https://cbrain.ca/getstarted.html	https://forum.cbrain.mcgill.ca/top?period=all
	VIP	https://vip.creatis.insa-lyon.fr	https://vip.creatis.insa-lyon.fr/documentation/	https://github.com/virtual-imaging-platform/VIP-portal/issues
	brainlife.io	https://brainlife.io	https://brainlife.io/docs/	https://brainlife.slack.com/
	ChRIS	https://app.chrisproject.org	https://chrisproject.org/docs	https://matrix.to/#/#chris:fedora.im

The platforms in each section or subsection are arranged chronologically by their publication date, and a table summarizes important usage information for each platform in the appendix of this review. We examined each platform’s installation and documentation, and launched a simple but informative use-case to evaluate their capacity to support investigations of analytical flexibility. In all sections except Section 4 (Numerical Stability), we focused on determining whether skull-stripping (also referred to as brain extraction), a common neuroimaging preprocessing step, could be performed successfully using two distinct tools (any two) on the single-subject test dataset ds005072 from OpenNeuro. The flexibility to perform skull-stripping with any two tools, rather than a fixed pair, reflects the varying availability of tools across platforms. Section 4 takes a distinct approach, focusing on the numerical stability of skull-stripping operations within FreeSurfer. This focus arises because the numerical stability platforms reviewed here aim to simulate the impact of numerical errors introduced by environmental variability within a given tool rather than exploring variability across tools, as emphasized in the other sections of this review.

Our findings, based on both objective and subjective criteria, are comprehensively discussed and summarized. Table 2 provides an overview of the categories, criteria, and methodologies employed to evaluate the platforms in this review. In this context, the term “errors” specifically refers to *software errors*—defined as errors that prevent the tool from being set up or executed—rather than processing errors, where the tool produces incorrect outputs. All tests were conducted by a single experienced tester, with confirmation by a second tester if the initial attempt was unsuccessful. While this approach is limited in scope and does not represent a broad trial across multiple testers and systems, the potential for bias is mitigated by the use of well-defined evaluation criteria.

We do not discuss solutions for result variability stemming from test–retest reliability (Noble et al., 2021), population sampling (Marek et al., 2022), or image acquisition site effects (Han et al., 2006; Jovicich et al., 2006; Takao et al., 2011), which have been previously documented. Similarly, while we recognize the importance of leveraging a wide variety of representative data in evaluating software variability, data management is separate from neuroimaging pipelines and falls outside the scope of our focus on analytical variability stemming from tools and environments. Tools such as COINS (Landis et al., 2016), COINSTAC Vaults (Martin et al., 2023), LORIS (Das et al., 2011), XNAT (Klein et al., 2015), Flywheel (Tapera et al., 2021), or others play an essential role in managing large-scale imaging data, facilitating data sharing, and ensuring broader reproducibility. These tools are invaluable for addressing challenges related to study planning, data dissemination, and metadata annotations (Niso et al., 2022), but their evaluation is not directly related to our objectives.

**Fig. 3.**
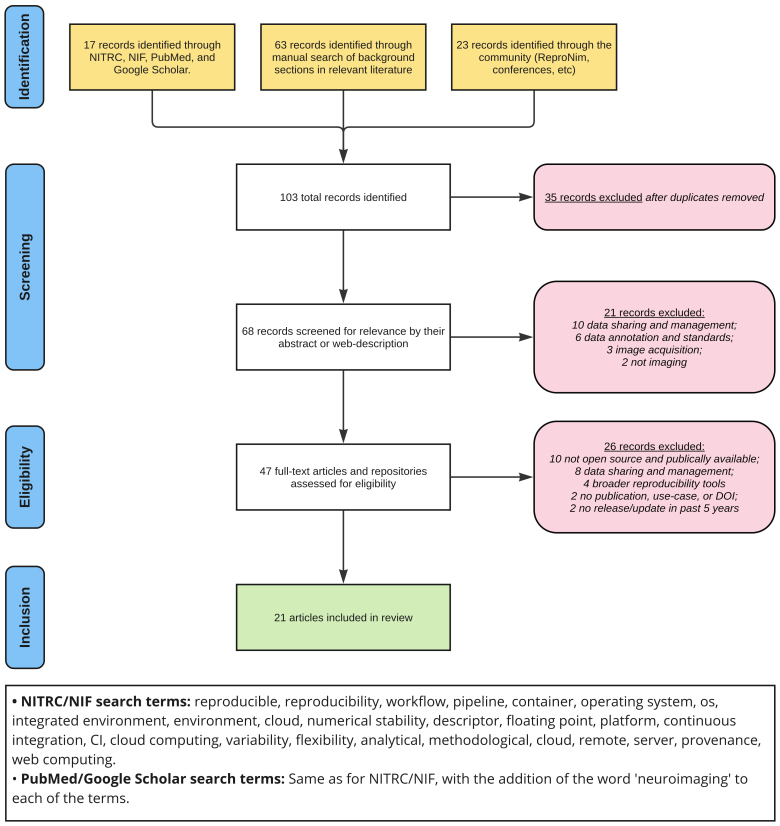
The identification, screening, eligibility, and inclusion process for tools featured in the review, with search terms for the search engines.

The inclusion criteria we use to incorporate platforms in this review are (1) at least one publication or DOI exists describing the platform, (2) the platform is open-source and publicly available, (3) there has been at least one release or code repository update in the past 5 years, and (4) at least one published neuroimaging study has used the platform. The platforms were found through an assortment of queries in the NeuroImaging Tools & Resources Collaboratory (NITRC (*NITRC: Welcome*, n.d.) and Neuroscience Information Framework (NIF) (Gardner et al., 2008) databases—two reference databases for neuroinformatics resources—, PubMed, Google Scholar, the background information sections of relevant papers, and suggestions and presentations from the neuroimaging reproducibility community (*Contact—ReproNim*, n.d.). Figure 3 shows the selection process for platforms featured in the review.

## 2 Tool Variability

Neuroimaging researchers encounter numerous tools with similar purpose and capabilities, which can produce different results when applied to the same data (Bhagwat et al., 2021; Botvinik-Nezer et al., 2020; Schilling et al., 2021). Given the absence of a single clear reference tool for most research questions and available data, verifying the result robustness across multiple tools is increasingly recommended. This section highlights platforms that can greatly accelerate the process of obtaining results across multiple different neuroimaging tools.

### 2.1 Pipeline containerization and tool repositories

Re-executing computational experiments is often challenging and time consuming. Sharing, installing, and running required tools across different environments are complicated by software dependencies such as libraries and settings, which can impact neuroimaging results (Glatard et al., 2015). In the past decade, software containers have emerged as a solution, allowing researchers to encapsulate applications and dependencies in lightweight, portable environments. Containers enable consistent and efficient deployment across different computing environments, regardless of the underlying OS. This simplifies tool distribution and execution, making it easier to test multiple pipelines across versions and environments. The software or platforms summarized in this section are listed in Appendix Table A1.

Understanding containerization is crucial before delving into other technologies covered in this section. Firstly, we note that containers are not the same as virtual machines (VMs). Virtual machines are complete OSes running on top of a physical host machine and emulating a full hardware environment. Containers share the host system’s kernel—the core part of the operating system that manages hardware resources and handles communication between hardware and software. This allows containers to have faster startup times and more efficient resource usage than VMs.

Docker (Merkel, 2014) is a popular container engine—a software that runs and manages containers. Users interact with the Docker client, which communicates with the Docker “daemon,” a background process that continuously runs to manage container operations such as building, running, and monitoring containers. Docker uses “images” as templates for containers, where an image is a lightweight, standalone package that includes an application and all its dependencies. Additionally, Docker provides the Docker Hub platform to find and share container images that can be used without installation. While Docker is versatile with a large support ecosystem, Singularity/Apptainer (Kurtzer et al., 2017) is preferred for high performance computing (HPC) systems. Singularity runs containers as regular applications rather than relying on a daemon, enhancing compatibility with HPC resource managers. Unlike Docker, Singularity does not require root privileges; rather it executes containers with the same permissions as the user on a host system, reducing potential vulnerabilities for HPCs. Singularity can use Docker images. Other container technologies, such as Podman for enhanced security in multi-user environments or LXD for workflows requiring full Linux systems, are not yet widely used in neuroimaging but could offer tailored optimizations and broaden the available tools for researchers.

#### 2.1.1 Container-based solutions

Container engines have been used to develop platforms and repositories containing many neuroimaging tool containers, further facilitating their findability and reuse (Sandström et al., 2022).

BIDS Apps (K. J. Gorgolewski et al., 2017) is a tool repository offering container images for multiple common ready-to-use neuroimaging pipelines. The containers process input data that are formatted as per the Brain Imaging Data Structure (BIDS) (K. J. Gorgolewski et al., 2016). The BIDS Apps website provides links to Docker Hub for downloading containers. All BIDS App containers share a common subset of command–line arguments, uniformizing their usage. Users can create BIDS Apps from a template and submit them for official inclusion. Additionally, the tools have Docker Hub tags corresponding to different versions, simplifying the testing of different versions of the same tool—an important feature in the context of analytical flexibility (Gronenschild et al., 2012).

To evaluate the ability of BIDS Apps to facilitate skull-stripping across multiple tools, we utilized Docker to run ANTs Cortical Thickness 2.2.0 (bids/antscorticalthickness:v2.2.0-1) and FreeSurfer 7.4.1 (bids/freesurfer:7.4.1-202309) images. These tools were executed on our single-subject test dataset by modifying the template “participant-level mode” commands specified in the README files of their respective GitHub repositories, which are linked on the main BIDS Apps website. The FreeSurfer container ran recon-all with the “—stage” parameter set to “autorecon1,” and ANTs was run with the “—stage” parameter set to “brain_extraction.” The computations ran without error, successfully producing two distinct brain extractions. The use of BIDS Apps streamlined the process by eliminating the need for installation and manual configuration of data locations, making it faster and more user-friendly to execute new tools.

Neurodocker (Kaczmarzyk et al., 2023) is a command–line program for generating custom neuroimaging Docker or Singularity containers. It simplifies the process of creating new containers that incorporate the applications, versions, and environments specified by users. Neurodocker offers many common neuroimaging tools, and generates a template file that users can modify to add new applications. Neurodocker can reduce container size by pruning non-essential files, a process called minification. For example, a complete Advanced Normalization Tools (ANTs) 2.0.3 image occupies 2 GB, but a “minified” version including only “antsMotionCorr” is 0.4 GB. Neurodocker is offered as both a Docker image and a Python API.

We tested the Neurodocker 0.9.5 Docker image using its command–line interface, as described in the documentation. Docker commands from the “examples” page were copied and executed to generate Dockerfiles for FreeSurfer 7.4.1 and FSL 6.0.7.1, a process that occurs almost instantaneously. Subsequently, we built the images using the provided commands and ran them with Docker, executing recon-all autorecon1 for FreeSurfer and BET for FSL on our single-subject test dataset’s T1 NIfTI file. Both brain extractions completed without error. The streamlined installation and container generation process significantly simplified access to these tools compared with traditional installation methods. However, it is important to note that further customization or debugging of Neurodocker configurations may pose challenges for less experienced users due to the level of familiarity required with container technology and command–line interfaces. That said, the documentation has helpful example commands for those exploring advanced features such as minification.

Neurodesk (Renton et al., 2024) provides a repository of containerized neuroimaging tools in a single environment. It offers a command–line interface as well as a virtual desktop environment with a graphical user interface (GUI), providing equivalent functionality on local, HPC, and cloud infrastructures. The GUI allows researchers without technical expertise to access the containerized tools. There are 99 “Neurocontainers” listed on their GitHub repository, and users can contribute their own. Neurocontainers are available for download from Docker Hub, DataLad (Halchenko et al., 2021), and the CernVM File System (CVMFS) (Blomer & Fuhrmann, 2010). CVMFS allows remote software access without local installation, transmitting only necessary files to minimize storage needs.

We installed Neurodesktop 1.7.0 on a Linux computer using the NeuroDesk-App .deb file provided on the platform’s website. We did not require documentation to use the platform. To access the environment, we connected to the “https://play-america.neurodesk.org” remote server, one of several available options. A window prompted us to log in via GitHub for identification, after which a containerized version of the Ubuntu OS with a full graphical user interface (GUI) was launched. Within the OS, a list of Neurocontainers was displayed in the applications menu, and selecting one automatically opened a command–line interface and configured it for use. We dragged and dropped the T1-weighted NIfTI file from our single-subject dataset into the containerized Ubuntu GUI and successfully executed commands for FreeSurfer 7.4.1’s recon-all autorecon1 and FSL 6.0.7.1’s BET on it. There is an option to delete user data from the server upon session exit. However, users should exercise caution when transferring private data to remote servers, unless they have created their own secure Neurodesk server. Using the offered “play” remote servers limits the amount of data that can be uploaded and the available CPU and RAM available, which may restrict the size and complexity of analyses. Instead, if users have Docker or Podman installed on their system, they can launch a local version of Neurodesk on their computer, which keeps all data and processing local. Further, advanced users may opt to host their own cloud-based version of Neurodesk.

NeuroDesktop provided quick access to a wide range of neuroimaging tools without requiring their installation, and with minimal command–line navigation. The underlying Docker Neurocontainer collection is a standout feature for programmers: it is well-maintained, documented, and extensive.

#### 2.1.2 Container descriptors

A container “descriptor” or “specification” is a file that includes information on how to run a tool in a containerized environment. A descriptor’s goal is to facilitate the integration of tools into workflow systems and to make them easily discoverable and reusable. Descriptors define a containerized tool’s usage by specifying valid input/output files, commands, execution parameters, and the underlying OS, tool version, and libraries. A descriptor may reference a container source (such as a Docker or Singularity recipe) but it does not build or define the container itself. Restricting the container’s usage to a specified scope ensures standardized and transparent operation, which, in turn, facilitates the validation of processes and simplifies their reproduction. This approach is particularly beneficial for automated tasks such as Continuous Integration (see Section 2.3) and complex workflows.

Boutiques (Glatard et al., 2018) is a framework for managing and describing command–line interfaces such as containers, primarily used in neuroimaging (e.g., Nipype (K. Gorgolewski et al., 2011), VIP (Glatard et al., 2013), CBRAIN (Sherif et al., 2014)). Boutiques abstracts container execution through JavaScript Object Notation (JSON) descriptors that describe the command–line and link to Docker and Singularity/Apptainer images. Researchers can also create or search for existing descriptors of containerized tools. Boutiques executes computations using consistent commands, making it easier to organize computations across multiple containers. It is available as a command–line utility and a Python package, and Boutiques-described tools are compatible with various infrastructures (personal machines, servers, clusters, cloud) as containers. Boutiques simplifies downloading descriptors for many ready-to-use containerized neuroimaging tools via Zenodo (*Zenodo*, n.d.). The Canadian Open Neuroscience Platform (CONP) ecosystem (Poline et al., 2023) wraps their extensive catalog of neuroimaging tool containers in uniform Boutiques descriptors.

Compared with manual procedures, Boutiques simplifies tool management by automating key steps. JSON descriptors have corresponding invocation files specifying the concrete parameter values. This eliminates the need for manual configuration of command–line parameters, reducing error and improving reproducibility. They also standardize parameterization in automated pipelines, enabling easy integration into larger workflows. The centralized availability of descriptors on platforms such as Zenodo reduces the effort needed to find, download, and configure tools. Boutiques also ensures compatibility across various infrastructures, avoiding the need for manual adjustments to execution commands.

We locally installed Boutiques 0.5.25 using pip (the standard tool for installing and managing Python packages from the Python Package Index repository), and followed the “Getting Started” notebook tutorial linked on the website main page. Using the command–line API, we searched for Zenodo IDs associated with FSL BET 6.0.1 (Zenodo ID 3267250) and FreeSurfer recon-all 7.1.1 (Zenodo ID 4043546)—tools previously described and published by the Boutiques community. For each tool, we generated an “invocation” JSON file, and refined the parameter fields manually in the invocation. The tools were then executed, yielding a successful skullstripping on our test T1 image. Boutiques simplified the process of acquiring, configuring, and executing the tools compared with manual procedures. Not having to explicitly specify container file bindings added a level of convenience, even for an experienced tester. However, not all neuroimaging tools come with readily available descriptors, which means users may need to define them from scratch. Creating JSON descriptors for complex tools may require a good understanding of Boutiques’ schema and command–line syntax, which could present a learning curve for new users. Alternatively, users can utilize the importer tool, which supports importing old descriptors, BIDS app descriptors (K. J. Gorgolewski et al., 2017), CWL descriptors (Crusoe et al., 2022), or docopt specifications (*Docopt*, n.d.). Additionally, users can take advantage of NiWrap’s extensive collection of over 1500 descriptors (https://github.com/childmindresearch/niwrap) which are based on the Boutiques Descriptor Schema.

#### 2.1.3 Integrated computational environments

Some researchers prefer running processings directly on their systems without using containers. They may find it simpler or lack the skills to manage containers. These researchers benefit from comprehensive computational environments with pre-installed tools and dedicated package managers for acquiring new software. These environments can be installed on their machines or run as VMs, providing access to many tools and a new environment to investigate software variability.

NeuroDebian (Halchenko & Hanke, 2012) is an integrated neuroimaging environment based on Debian Linux, offering over 750 open-source neuroscience tools easily installed via the standard “apt-get” command. The NeuroDebian repositories are available as command–line add-ons for multiple different OSes, and NeuroDebian is available as an installable or runnable image, including a Docker container.

The NeuroImaging Tools & Resources Collaboratory Computational Environment (NITRC-CE) (*NITRC: Welcome*, n.d.) is a VM image based on NeuroDebian, adding extra neuroimaging tools for custom or commercial cloud infrastructures.

Our evaluation of NeuroDebian yielded a varied experience. We followed the instructions in the “Get NeuroDebian” section of the NeuroDebian website’s front page. Integrating the NeuroDebian repository on a personal machine with Ubuntu 18.04 allowed a straightforward installation and use of FSL Bet 5.0 (the fsl-5.0-core package) to perform skullstripping on our test T1 subject. The python3-nilearn package displayed an “unable to locate package” error, which we learnt was due to package incompatibilities with Ubuntu 18.04. To overcome this, we create a Docker container with Ubuntu 16.04. In this container, we successfully installed NiLearn 0.2.5 and used the compute_epi_mask, apply_mask, and unmask functions in Python to perform brain extraction. We encountered similar issues with the official NeuroDebian 0.42.1 Docker image on Docker Hub, where we received “unable to locate package” errors when trying to install both tools, presumably also due to OS compatibility issues. It is important to verify package–OS compatibility on the NeuroDebian website to ensure the tools can be used in their preferred environment. We note that maintaining and updating over 750 packages across multiple OSes require huge ongoing effort, which is often done voluntarily (Westner et al., 2025).

Another project with a similar approach is NeuroFedora (“29th Annual Computational Neuroscience Meeting,” 2020), which offers various neuroimaging tools among a broader neuroscience toolset. The NeuroFedora community packages, tests, documents, and integrates tools—there are currently 353 listed—in the Fedora Linux distribution. The tools are included in Fedora’s default package manager “DNF,” and can therefore be searched and installed on any derivative of the Fedora OS. A ready-to-use OS image includes 36 preinstalled tools, with others accessible via DNF.

We downloaded the “Fedora Comp-Neuro Lab 41” ISO image linked in the NeuroFedora website, and ran it using VirtualBox 7.0. The image enabled us to install Fedora 41 with Gnome OS, giving us access to the repositories where we could easily search and download the collection of neuroscience tools. We successfully installed and used Nilearn 0.11.1 through DNF using the command specified on the NeuroFedora documentation website, and used the compute_epi_mask, apply_mask, and unmask functions in a Jupyter Notebook to carry out a brain extraction on the test subject T1. We were not able to find a second tool in the repository to perform a brain extraction. The current neuroimaging selection of tools is limited (13 tools) in comparison with the extensive computational neuroscience data analysis, simulation, and utility selections.

### 2.2 Workflow engines

Neuroimaging data processing typically involves scripts and commands that execute software modules on input data to generate image derivatives, numerical results, and visualizations. These processing “workflows” are often repetitive to program and can include complex sequential or parallel steps with multiple components. Complex workflows require significant amounts of effort to describe, share, and set-up for re-execution in different environments. Workflow engines provide a machine-readable way of encapsulating and re-executing workflows in different environments, ensuring reproducibility and provenance by constraining analytic choices such as tool versions and execution order. They also enable researchers to swap or iterate modules to investigate the effect of analytical choices. The workflow engines summarized in this section are specifically designed for neuroimaging and are listed in Appendix Table A2. For a larger overview of the state of workflow engines beyond neuroimaging see da Silva et al. (2023).

All-but-one of the tools in this section are based on Directed Acyclic Graphs (DAG) implementations. A DAG is a data structure consisting of nodes connected by directed edges, without any cycles. In workflow engines, nodes represent tasks (tool executions), and edges encode dependencies between tasks or ordered steps within a workflow. The absence of cycles in a DAG ensures that workflows progress in a well-defined order, avoiding circular dependencies that could lead to ambiguous or endless execution. This structured representation offers benefits such as simplifying the scheduling of parallel computations for efficiency during workflow execution. It also simplifies recording provenance, and using node completion statuses as “checkpoints” helps to avoid recomputation when re-launching workflow steps. The absence of cycles in a DAG is also important for facilitating the comparison of outputs from multiple tools. By maintaining a clear, sequential structure, it enables straightforward evaluation of results from different tasks and ensures that dependencies between tools are transparent. This clarity is crucial when comparing outputs across various tools, as it minimizes the risk of ambiguous results, which can occur if cycles were present.

However, DAGs may struggle to efficiently represent workflows with dynamic or iterative dependencies, requiring additional workarounds or alternative approaches for such scenarios. Additionally, DAG-based workflows can require significant storage for intermediate data and metadata, particularly in large or complex workflows.

Nipype (K. Gorgolewski et al., 2011) is a very popular neuroimaging workflow engine, implemented as a Python library. Its unified interface to numerous neuroimaging tools allows users to execute any tool in a similar manner in their code. Nipype workflows are shareable and re-executable as Python scripts, and can be executed locally or remotely on clusters.

“Interfaces” are at the core of Nipype, acting as bridges between Python code and external neuroimaging tools. Interfaces specify input parameters, outputs, and dependencies for use in a DAG node. Users can define custom interfaces or choose from a wide range of existing interfaces listed on the documentation website.

We installed Nipype 1.9.2 via pip. Following the documentation quickstart tutorial, we imported the pre-made FSL BET and FreeSurfer ReconAll interfaces. These executed our locally installed versions of BET (6.0) and FreeSurfer (7.4.1) on our test dataset’s T1 image. We integrated nodes for BET and ReconAll (using the “autorecon1” directive) in a workflow, and executed it using the “MultiProc” workflow runner, which distributes the two tasks across multiple cores. The two brain extractions were successful. Note that Nipype—while active and functional—is being superseded by Pydra (Jarecka et al., 2020), discussed later in this section.

Numerous notable open-source reproducibility-related pipelines use Nipype workflows in their backend. The Configurable Pipeline for the Analysis of Connectomes (C-PAC) (Cameron et al., 2013) uses Nipype to carry out connectivity analyses for resting-state fMRI data. It automates pre-processing and analyses across multiple different pipelines to assess their impact on results. C-PAC also offers a GUI and a BIDS App container.

Similarly, Clinica (Routier et al., 2021) and ClinicaDL (Thibeau-Sutre et al., 2022) aggregate and harmonize various commonly used neuroimaging analysis and pre-processing methods as Nipype workflows in a unified framework. Clinica also incorporates data management functionalities and is primarily operated through the command–line.

Pydpiper (Friedel et al., 2014) is a Python-based workflow engine specializing in image registration workflows—the transformation mapping one scan into the space of another. Pydpiper was written around four specific use-cases: iterative groupwise registration, chained adjacent time point registration, longitudinal two-level registration, and automated multi-atlas label generation. It offers out-of-the-box registration pipelines (mainly leveraging ANTs and MINC toolkits) with customizable modules and parameters. Advanced users can modify toolkit templates to integrate new tools. Pydpiper can execute external commands via Singularity/Apptainer, and can execute workflows locally, as well as servers and clusters.

Pydpiper’s GitHub repository recommends running it via the Mouse Imaging Centre (MICe) Singularity/Apptainer container, and it can also be installed from the source code as a Python package. We encountered a “metadata-generation-failed” error while using pip to install Pydpiper (version “2.0.19.1”). We also attempted versions “2.0.9” and “1.9,” but they were unavailable. Manual installation resulted in an “AttributeError: module ‘collections’ has no attribute ‘MutableMapping’,” and the container option was non-functional. We could not find documentation on the MICe wiki and were, therefore, not able to properly evaluate Pydpiper (the last updates are from 2022).

Automatic Analysis (AA) (Cusack et al., 2015) is a MATLAB-based workflow engine offering tools for various imaging tasks across modalities. It is available on Linux systems (requiring MATLAB) or as a containerized BIDS App. Users create workflows through a MATLAB userscript specifying inputs, outputs, settings, and parameters, alongside a tasklist XML file describing module order. AA offers 579 modules—many of which leverage SPM, FSL, and FreeSurfer to perform tasks—and provides customizable userscript and tasklist templates. Modules generate diagnostic results with plots and measures for quality control.

We evaluated AA locally by pulling the BIDS App for AA v0.2.0 (bids/aa), which packages all dependencies in the container, allowing users to run it without requiring a MATLAB license. While we were ultimately able to successfully perform the brain extractions, the process proved to be complex and time consuming, involving extensive troubleshooting. Upon initial use, following the BIDS App’s GitHub documentation README, it failed to function as expected. Despite providing our inputs, it would only run the example “BIDS_tasklist.xml” and corresponding “BIDS_aa.xml” parameters, providing no error messages to help understand its behavior. To overcome this, we bypassed the documented parameter usage and injected our custom XML files, replacing the “/opt/BIDS_aa.xml” and “/opt/BIDS_tasklist.xml” within the container. The FreeSurfer license file required a similar procedure. Constructing a working tasklist was not intuitive. While some documentation exists on AA’s GitHub Wiki, it lacks in-depth explanations of the hundreds of modules and the correct sequence in which to apply them (FSL and FreeSurfer require specific initialization modules). To determine the appropriate configuration, we had to browse examples and read individual module MATLAB and XML files on AA’s GitHub repository. After considerable trial and error, we successfully employed the “aamod_freesurfer_autorecon” and “aamod_bet” modules on the test dataset T1 image. Creating new modules (DAG nodes) is also more complicated than in the other platforms in this section as it involves interacting with AA internals—the process is documented for MATLAB programmers on AA’s GitHub Wiki. We note that AA’s BIDS App GitHub repository was archived in January 2024. As of February 2025, the container we used is not listed on the BIDS Apps website, and AA’s official website is not available.

Fastr (Achterberg et al., 2016) is a workflow engine that claims to function on “*virtually any image archive and processing infrastructure*”. It implements a plugin-based design; tools, data types, and the execution engine are integrated as extensible plugins. Fastr also supports simultaneous versions of the same tool in a workflow.

Fastr is a Python library. Tools are specified as XML or JSON files, with a schema describing inputs, outputs, data types, execution settings, and metadata. The execution plugin checks tool input and output data type compatibility before launching computations, and works locally or on a cluster.

We used pip to download Fastr 3.3.1. We followed their documentation’s “quick start guide.” We encountered errors while creating a “source node” for the DAG. When we attempted to run Fastr 3.1.4 instead, an error occurred upon package import. We tried using different versions of Python (3.10.10, 3.6.0, 3.6.15, and 2.7.17) and ran into errors during importing or installation. Unlike Nipype or AA, we could not find any neuroimaging tool specifications included in the Fastr tools.

Nextflow (Di Tommaso et al., 2017) is a workflow engine widely used in bioinformatics, with variations such as TractoFlow (Theaud et al., 2020) and TIGR-PURR (*TIGRLab Pipelines: Utterly Reproducible Research (TIGR-PURR)—Tigrlab_nextflow Documentation*, n.d.), which are adapted for neuroimaging. It operates on standard systems (Portable Operating System Interface (POSIX)-compatible Linux, OS X) and targets researchers with programming experience. Nextflow simplifies interactions with various schedulers, that is, software that automate and manage the order and timing of tasks, web computing platforms, and resource scaling.

Nextflow uses a “dataflow” approach instead of a DAG, where computations run when input requirements are met, reducing storage needs for precomputed graphs. Users must learn Nextflow’s custom “Domain-Specific Language” (DSL) syntax and environment for defining workflow sequencing.

We installed Nextflow 24.10.4 following the website’s instructions for command–line. We created two scripts roughly following the functionalities laid out in their “Fundamentals Training” documentation. One script included a process performing skullstripping using a local installation of FSL BET 6.0, and the other using a local installation of FreeSurfer recon-all (autorecon1) 7.4.1. Both scripts ran successfully. While we only scratched the surface of Nextflow’s capabilities with these tests, there is a learning curve to using Nextflow due to their custom syntax. Based on our experience, aside from the tests discussed here, we have noticed a few minor limitations with Nextflow: Nextflow’s invocation of Singularity/Apptainer hindered our ability to add specific mounts and overlays, and their outputs relying on symbolic links to arbitrary absolute paths were an inconvenience for moving or archiving the outputs.

Nipype’s follow-up is a software ecosystem centered around Pydra, a general scientific workflow engine (Jarecka et al., 2020). Pydra alleviates some of Nipype’s limitations: it is more lightweight and provides simpler means for incorporating workflow components in different formats (Python, command–line, container, etc.). It also implements nested DAGs, allowing greater re-use of computations, and providing output recombination and summary features (similar to the Map-Reduce pattern). Workflow execution can occur locally, or on HPCs through execution managers such as Dask, “Sun Grid Engine” (SGE), and the “Simple Linux Utility for Resource Management” (SLURM), with flexible computing resource scaling. Pydra is available as a Python package.

We downloaded Pydra 0.25 using pip, and followed the GitHub repository tutorial notebooks. We created a single Python script that used ShellCommandTask “Tasks” to create and execute our local installations of FSL BET 6.0 and FreeSurfer recon-all 7.4.1 (autorecon1). The skullstrip of the test dataset T1 image was successfully performed. We noticed several pip packages that adapt popular neuroimaging tool interfaces for Pydra (e.g., pydra-fsl, pydra-freesurfer, pydra-ants). However, we found Pydra intuitive enough to use directly, so we did not need these adaptations. Pydra is still under development, but we found it easier to use and is more flexible than Nipype as we could use Python code, command–line instructions, and containers without importing or defining interfaces.

### 2.3 Continuous integration

Continuous Integration (CI) is standard in software engineering, involving automated tests launched in a container whenever new code is committed to a code repository. These tests, written by developers, ensure the software functions correctly with each update to the codebase.

In scientific research, CI has been brought up as a strategy to automatically evaluate and facilitate reproducibility in computational experiments (Beaulieu-Jones & Greene, 2017; Krafczyk et al., 2019). Scientific findings are rarely updated post-publication, even as the tools and datasets used evolve. In contrast, CI allows researchers to frame a result produced by their code as a “test,” and keep track of how the result is affected by updates in the underlying codebase. Several platforms provide free CI services for code repositories, such as Jenkins, CircleCI, TravisCI, GitLab, and GitHub Actions. However, these platforms are not well suited for neuroimaging experiments, as they lack the ability to handle high-compute workloads and the privacy-sensitive data often required in such analyses.

NeuroCI (Sanz-Robinson et al., 2022) leverages CI to investigate analytical flexibility in neuroimaging experiments. It is a Python framework that launches distributed computational experiments across multiple pipelines and datasets, and can continuously ingest new pipelines and datasets. Given the large computational resource and privacy needs of neuroimaging analyses—which are beyond the scope of traditional CI services—computations are offloaded to an HPC platform (see Section 5). A dashboard lets users visualize provenance and results in their browsers, observing experiment result robustness and replicability as methods and data change.

NeuroCI is used by cloning the GitHub repository and following the README instructions. We evaluated the “v1” tag in the “old-version-cbrain” branch. Setup requires significant manual configuration. Integration requires accounts for CircleCI, GitHub, CBRAIN, and HPC systems, along with their respective configurations, as well as configurations for NeuroCI and the desired CBRAIN tools. Managing accounts, permissions, and automated computations on private data may challenge users unfamiliar with distributed systems. The setup of a functional CBRAIN Data Provider was not documented in the README, leading to delays in configuring permissions and identifying the requirement for a server without two-factor authentication (2FA) for data ingestion. After this, we were able to successfully submit and execute our brain extraction tasks (BET 5.0.9, FreeSurfer 7.4.1).

NeuroCI is still in its early stages; documentation remains sparse, and unit tests have yet to be implemented. Additionally, as with any platform that relies on multiple distributed dependencies, NeuroCI is susceptible to failure points such as API incompatibilities, data format changes, and evolving authentication protocols if not actively maintained. A new version based on the Nipoppy framework (Nipoppy contributors et al., 2025) is actively being developed in the “master” branch to simplify setup, reduce required accounts and components, and enhance resilience to external changes. Notably, this update will interface directly with HPCs, eliminating the CBRAIN dependency, and replaces CircleCI with GitHub Actions for CI. A summary of NeuroCI is provided in Appendix Table A3.

### 2.4 Summary and guidance

Neuroimaging researchers can select container-based solutions for their analyses based on their specific requirements: BIDS Apps provide ready-to-use, standardized pipelines with minimal setup, ideal for researchers seeking straightforward workflows compatible with BIDS-formatted datasets; Neurodocker supports developers and advanced users requiring customizable, lightweight containers; and Neurodesk offers an extensive container repository, with the versatility of a command–line interface for technical users and a GUI that lowers barriers for non-technical users. Users with limited technical expertise may benefit most from Neurodesk’s GUI, while those prioritizing efficiency and flexibility in creating customized containers might prefer Neurodocker.

Boutiques further supports workflow standardization by enabling consistent parameterization and integration of containerized tools into automated pipelines through the use of descriptors.

For researchers who prefer avoiding containers, environments such as NeuroDebian and NeuroFedora offer pre-installed tools and extensive repositories but require familiarity with specific Linux-based operating systems. NeuroDebian offers a larger repository of neuroimaging-specific tools, while NeuroFedora’s repository is better equipped for broader neuroscience analyses. NeuroDebian and NeuroFedora can also be used to build container images.

Workflow engines play a crucial role in enabling analytical flexibility by automating the execution of sequences of computations, enabling the exchange of equivalent modules, and ensuring reproducibility and scalability across different environments. Nipype is a great choice for researchers who want a widely used, robust, Python-based system with a wide array of pre-built interfaces for popular neuroimaging tools, and is ideal for both beginners and experienced users. However, for those seeking a more lightweight and flexible solution, Pydra offers a simpler Python-based interface with support for nested workflows and resource scaling, making it a better choice for advanced users working with complex workflows. Nextflow is also well-suited for those needing scalability and advanced resource management, but comes with a steeper learning curve due to its custom syntax. For researchers using MATLAB, Automatic Analysis (AA) offers compatibility with many popular neuroimaging tools, though it involves a more complex workflow creation process and presents challenges when working with the archived BIDS App container.

NeuroCI is designed to continuously evaluate and compare neuroimaging results across multiple pipelines and datasets, accommodating the addition or growth of datasets and the integration of new containerized pipelines. However, the framework is still under development and is currently best suited for advanced users.

## 3 Data Provenance

### 3.1 Data provenance platforms

Derived datasets often lack provenance documentation, which records how files were transformed through computations. This practice is rare in neuroimaging literature, yet knowing a dataset’s precise version after cleaning, standardizing, and preprocessing is crucial for reproducing and interpreting scientific results. The absence of provenance can lead to irreproducible analyses, difficulty in verifying results, and the inability to trace and correct errors or biases introduced during data processing. Numerous studies have highlighted that lack of provenance in neuroimaging computational workflows can hinder the reproduction of scientific findings (MacKenzie-Graham et al., 2008; Nichols et al., 2017; Poline et al., 2012). Exhaustively and transparently tracking a dataset’s evolution ensures results can be re-obtained and compared across studies, thus promoting scientific rigor. The platforms in this section simplify tracking dataset transformations, and are listed in Appendix Table A4.

DataLad (Halchenko et al., 2021) is the primary tool for the neuroimaging community’s data versioning needs. DataLad allows users to download or create new datasets—and tracks all the changes the dataset undergoes. While useful in many domains, its development and use-cases were initially targeting neuroimaging projects. DataLad is available as a Python library and a command–line tool.

DataLad adapts a Git workflow for open-source software development to datasets, facilitating collaboration and distributed dissemination. However, since Git is not suited to handling large or binary files, Datalad uses the git-annex (*Git-Annex*, n.d.) data logistics system to track files without storing them directly in a Git repository. Instead, a reference to the file (based on a checksum of its content) is added to the repository, enabling the file to be found and fetched from its web-based storage location. Changes to datasets are tracked via uniquely generated Git tags, ensuring accurate versioning. DataLad’s website indexes numerous available datasets, many of which are open and useful for neuroimaging researchers.

A key feature of DataLad in the context of analytical variability is the datalad “run” command, which allows users to execute arbitrary shell commands and record their impact on a dataset. When executing a command with “run,” DataLad creates a new Git commit, recording the command itself, its arguments including input data, and any environment variables. Furthermore, it tracks the command’s outputs and how they modify the dataset, ensuring a complete record of each data transformation. This information, along with captured software dependencies, is stored as metadata within the Git history. This allows researchers to replicate the exact analysis by re-executing commands in the original order to reproduce the results.

We installed DataLad 0.2.3 via pip and used the “install” and “get” commands to retrieve the test BIDS dataset from OpenNeuro for evaluating the tools in this review. We followed the “DataLad, run!” section of the DataLad Handbook. An empty directory was initialized as a DataLad dataset using the “create” command, and within it, we used “run” to re-execute the commands and containers generated to test NeuroDocker from Section 2.1.1 on the T1-weighted image, specifically FreeSurfer “recon-all” 7.4.1 (autorecon1 stage) and FSL “BET” 6.0.7.1. The brain extractions completed successfully, and DataLad versioned the changes in the Git history, allowing us to trace the executed commands and generated files.

The BIDS App Bootstrap (BABS) (Zhao et al., 2024) leverages DataLad, BIDS Apps, and the FAIRly big framework (Wagner et al., 2022) for reproducibly analyzing datasets. Users provide BABS with BIDS DataLad datasets, a DataLad dataset indexing BIDS App containers, and a parameter configuration “YAML Ain’t Markup Language” (YAML) file. Both the BIDS Apps and datasets are tracked through Git, providing a thorough audit trail of processings. The FAIRly big framework organizes computations: files are cloned from permanent storage to a temporary HPC directory, where they are processed by the BIDS App. BABS is compatible with HPCs running SGE and SLURM schedulers, it ensures computations are parallelized where possible.

We installed BABS version 0.0.8 through pip on a SLURM HPC via Compute Canada. We followed the “example walkthrough” featured on the BABS documentation website, modifying it to run the BIDS Apps we successfully used in Section 2.1.1 (FreeSurfer 7.4.1 and antsCorticalThickness 2.2.0) on the test BIDS dataset. We initialized a BABS project for each BIDS App, and job submission to the SLURM scheduler on the HPC was straightforward and successful. However, the brain extractions failed. BABS generates Singularity commands using the —participant-label parameter, whereas the BIDS Apps we tested—and others we reviewed for brain extraction—use —participant_label instead, as stated in their GitHub repository READMEs. Additionally, these BIDS Apps specify that the participant label should not include the sub- prefix, unlike the format used by BABS (e.g., sub-01 in our test case). As a result, we encountered the error: “run.py: error: unrecognized arguments: —participant-label sub-01.” We were unable to find a configuration option to modify this behavior. This underscores the importance of verifying that the chosen BIDS Apps are compatible with BABS’ Singularity command generation. Additionally, we experienced a considerable runtime overhead in job submissions, likely due to the interaction between the FAIRly big framework and a heavily utilized Lustre file system on the HPC. However, this overhead may be less pronounced when working with large datasets that can fully leverage the framework’s parallelization capabilities.

### 3.2 Summary and guidance

DataLad is appropriate for data managers, neuroimaging researchers, and anyone needing robust version control and reproducibility in data and computational workflows. Its “run” command allows users to track dataset transformations and computational steps on both local machines and HPCs. DataLad is suited for those who require fine-grained tracking of data changes, command line executions, and dependencies.

BABS builds on DataLad’s functionality, making it specifically optimized for researchers working with BIDS datasets and BIDS Apps in HPC environments. It provides integration with SLURM and SGE schedulers, and benefits from parallelization to speed up computations. Setting up a BABS project, however, may be a laborious process for those unfamiliar with its dependencies, and scheduled computations may incur a runtime overhead. The user must also check their desired BIDS App is compatible with BABS singularity command generation.

## 4 Numerical Stability

Neuroimaging data processing algorithms typically use mathematical operations based on floating-point data types. Most current CPUs implement the IEEE-754 standard, which dictates how floating-point arithmetic must be performed, including the formats’ size, the operations, and rounding modes to ensure code portability across different architectures. Numerical errors in calculations—rounding errors, cancellation, over/under-flow errors, etc.—occur due to the finite precision of floating-point arithmetic, presenting themselves when values cannot be represented exactly within a given floating-point format. As a consequence, floating-point arithmetic is not associative, meaning that the order of operations impacts the result. The resulting instability can be triggered by data noise or computational environment factors such as OS changes, compiler versions, code modifications, library updates, and non-deterministic calculations due to parallelization (vectorization, task scheduler, asynchronous communications, etc.). The instabilities can lead to variances in result precision, computation performance, and error handling. The errors can propagate across modules in complex pipelines with sequential or iterative steps where each subsequent calculation depends on a previous one, and become amplified as each module contributes to the error. In neuroimaging, numerical instabilities can lead to reproducibility concerns and have been observed in popular tools such as FSL (Salari et al., 2021; Vila et al., 2024), Freesurfer, CIVET (Glatard et al., 2015), Dipy (Kiar et al., 2021), fMRIPrep (Chatelain et al., 2023), as well as registration and segmentation machine learning models (Pepe et al., 2023). This section features software used in neuroimaging research to quantify numerical instability impact. The software packages are listed in Appendix Table A5.

In particular, the software packages in this section all rely on Monte Carlo Arithmetic (MCA) (Parker & Langley, 1997). MCA is a technique commonly used in research and software development to investigate numerical instability by introducing controlled amounts of noise in the floating-point operations. By running many MCA repetitions where floating-point operations are perturbed, researchers can measure the sensitivity of their tools and models with regard to the floating-point arithmetic.

This section focuses on platforms designed to investigate the impact of numerical errors introduced by environmental variability within a given pipeline, employing a distinct testing methodology compared with the other sections of this review which explore variability across different tools. To assess these platforms, we instrumented FreeSurfer 7.3.1 using the featured platforms and encapsulated the modified software within a container. We then conducted a proof-of-concept test by running the recon-all command (autorecon1 stage) using this containerized, instrumented FreeSurfer. A description of the instrumentation process for each tool is provided in the respective tool sections. In a typical numerical imaging study, this process would involve multiple container runs with the instrumented FreeSurfer to generate replicate outputs, followed by statistical analysis to quantify the variability introduced by environmental factors. However, for the purposes of this paper, we limit our evaluation to demonstrating successful execution of the instrumented FreeSurfer within the container and the generation of expected outputs. We note that a Verificarlo developer with extensive experience in Verrou assisted us in setting up both tools for the tests in this section.

Verrou (Févotte & Lathuilière, 2016) is a software that is available as an optional add-on to the popular Valgrind memory debugger (Nethercote & Seward, 2007). Verrou dynamically replaces floating-point operations with alternative counterparts within Valgrind’s VM. Verrou operates directly on the tool’s binaries, avoiding the complexities of source code recompilation. It provides several alternative floating-point models (also called backends), including multiple variants of the MCA model, which users can select according to their needs. At the end of execution, Verrou reports the number and types of floating-point operations that have been instrumented. Additionally, the user can specify precise parts of the code to instrument, which saves processing time.

Testing Verrou, although successful, required a challenging and time-consuming installation process. Installation within a Singularity container necessitated careful configuration. After iterative trials with various Valgrind (3.19.0, 3.20.0, 3.21.0, 3.22.0) and Verrou (2.4.0, 2.5.0) versions, a successful setup was achieved with Valgrind 3.21.0 and Verrou 2.5.0. This was accomplished by following the instructions from the Verrou GitHub repository, utilizing the provided “make” file for installation. FreeSurfer 7.3.1 was subsequently installed within the same container. The recon-all command (autorecon1 stage) was executed under Valgrind, setting the tool parameter to “verrou” and the “rounding-mode” parameter to random. The output for our test T1 image resembled the regular FreeSurfer output directory and file structure.

Verificarlo (Denis et al., 2016) is a compiler that is designed as a plug-in extension to the popular LLVM compiler, and works on code written in C, C++, and Fortran. It compiles the code replacing all floating-point operations with a generic call, capturing the influence of compiler optimizations on numerical calculations. The user can then select the backend to execute at runtime, including MCA. After users compile their programs, they can simulate round-off noise or lower levels of precision. Verificarlo can instrument the entire code or instrument a single function of interest, and offers a Docker release alongside the source code. A limitation of Verificarlo is that users must independently compile the tools they wish to analyze, which can be tedious or even impossible for closed-source programs. For example, recompiling an entire neuroimaging pipeline can be particularly challenging.

To reduce overhead, Verificarlo integrates “fuzzy lib-math,” a framework that instruments elementary mathematical functions from the libmath library, obtaining an accurate approximation of environment-driven numerical variability for neuroimaging purposes (Salari et al., 2021). Verificarlo and fuzzy lib-math 0.9.1, and FreeSurfer 7.3.1 were added to a Docker or Singularity container using a multi-stage build, as instructed in the verificarlo/fuzzy GitHub repository README. The instrumented math library is copied into the target image. The LD_PRELOAD linker ensures the use of these instrumented libraries during execution. We set the virtual precision and instrumentation mode of Verificarlo with the command indicated in the MCA-libmath GitHub repository. Once finished, we could run FreeSurfer’s recon-all command (autorecon1 stage) without error, and the computations yielded the expected output structure directory and files for the skullstripped T1 image from our single-subject test dataset.

### 4.1 Summary and guidance

Choosing between Verrou and Verificarlo for neuroimaging numerical stability analyses depends on the trade-offs between setup complexity and execution speed. Both tools are designed for tool developers with advanced programming skills, not end-users. Verificarlo requires users to compile the tools they wish to study, which demands significant technical expertise but leads to faster execution once set up. Verrou, however, operates directly on binaries, simplifying the setup process but sacrificing performance due to its use of quad precision and serialized execution via the Valgrind VM, which also lacks support for AVX-512 instructions—potentially causing crashes when analyzing binaries compiled for CPUs that utilize these extensions.

## 5 Web Computing Platforms

### 5.1 Web computing platforms

A major challenge in neuroimaging research is providing adequate cyberinfrastructure for the data- and compute-intensive demands of modern imaging methods. To address this, web computation platforms have become widespread in neuroimaging centers. These platforms share common features designed to make large-scale computational analyses more accessible, especially for researchers lacking programming or server interaction skills.

The platforms discussed here are accessed via a browser, serving as an interface between users and a HPC or server. They allow users to interact with remote servers to access distributed data and perform computations using a GUI, shielding users from complexities of hardware provisioning, tool installation, scheduling, data transfers, and experiment scaling. Computational environments are automatically provisioned, enabling users to launch pre-configured containers or executables, or integrate new tools. Access to numerous pre-configured tools without setup reduces the barrier in investigating analytical flexibility. These platforms also facilitate easy sharing of workflows, data, derivatives, and visualizations, promoting transparent provenance and reproducibility. They also offer robust infrastructure that minimizes system downtime and implement redundancy to protect the user’s work against hardware failures.

Since the platforms featured in this section are all largely based on the functions described in the previous paragraph, we have summarized their distinct features and use-cases in Appendix Table A6. Despite differences in graphical interfaces and tool selections, testing the platforms was a comparable user experience. Users can sign up for an account and start using their portal page promptly, upload their own data (or use publicly available datasets), choose a tool, select input files and parameters, monitor computation completion progress, visualize results in the browser, and download the results locally. The platforms automatically took care of routing the computations to the correct computational resources. With CBRAIN (Sherif et al., 2014), VIP (Ferreira da Silva et al., 2011; Glatard et al., 2013), and brainlife.io (Hayashi et al., 2024), we had launched computations soon after logging in, and these finished successfully. We uploaded a single T1 scan to each platform. On CBRAIN we ran FSL BET 5.0.9 and FreeSurfer 7.4.1 to skullstrip the T1 input, on VIP we skullstripped it using FSL BET 6.0.1 and FreeSurfer 7.3.1, and on BrainLife we skullstripped it using FSL BET 6.0.1 and FreeSurfer 7.3.2. For ChRIS (Pienaar et al., 2015) we skullstripped the image using FreeSurfer 7.4.1 and MALPEM 1.3.0. We received assistance from the ChRIS developers to set up computations with FreeSurfer.

As in all cases of remote clinical data upload, researchers should ensure data compliance before remotely uploading clinical data to avoid privacy issues. Additionally, data privacy regulations, such as the General Data Protection Regulation (GDPR) in the European Union, can impose limitations on the use of web computing platforms for sharing and analyzing clinical data. These regulations require data anonymization protocols and may restrict data transfers for platforms hosted in countries with differing privacy standards. This adds complexity for researchers in international collaborations. It is, therefore, essential for users to verify compliance with institutional and legal data governance policies before uploading sensitive data to ensure that legal obligations are met.

We note that web computing platforms exhibit inherent complexity, particularly due to the frontend backend division and reliance on diverse third-party dependencies (i.e., libraries, frameworks, infrastructure services). This complexity can foster numerous points of failure, including API incompatibilities and data format changes, requiring considerable effort to maintain. The availability of storage and compute resources may also vary, impacting the user’s experience.

### 5.2 Summary and guidance

It can be difficult to choose between web computing platform infrastructures, as the core end-user experience and capabilities are quite similar despite varying implementations. All platforms offer seamless access to remote computational resources for running a broad selection of containerized neuroimaging tools, visualizing images, and feature user-friendly point-and-click GUIs that make them accessible to those without coding skills. The platforms all feature well-developed documentation pages.

In our experience with the main live instances of the web-apps for each platform, CBRAIN and BrainLife stand out for offering a more extensive selection of neuroimaging tools, making them particularly well suited for exploring analytical flexibility. We observed longer waiting times on BrainLife compared with other platforms. Configuring BrainLife to operate with custom compute resources is a viable option; however, it exceeds the technical capabilities of the majority of end-users who would benefit from the GUI. VIP, originally designed for medical image simulation, offers a more limited selection of widely used neuroimaging tools. Additionally, VIP restricts “beginner” accounts to a single active workflow per user. Users must contact administrators to upgrade their accounts in order to run multiple tools simultaneously. However, it is distinguished by a particularly simple and aesthetically pleasing interface compared with the other platforms. ChRIS provides a somewhat less intuitive interface (currently under active development), but offers significant flexibility in workflow customization: users can revisit their analyses at any time and add new tool (“*plugin*”) nodes to the computation DAG to run additional tasks as needed. Ultimately, the best platform for a user depends on their specific neuroimaging tool requirements (which can be reviewed on the platform’s web-apps).

Although these platforms are primarily designed for users who benefit from graphical user interfaces (GUIs), it is worth noting that CBRAIN, VIP, and BrainLife all offer APIs that allow users to interface with their services directly through code. Additionally, ChRIS is in the process of implementing functionality to drive analyses using the command line, as well as JavaScript and Python client libraries.

## 6 Summary and Take Away

This review is motivated by the increasing role that analytical flexibility plays in neuroimaging robustness. The large amount of methodological choices researchers are faced with can affect the results they obtain, and there is often no ground truth to determine which of the choices are optimal for their particular experiments. As the magnitude and ubiquity of the reproducibility issues posed by analytical flexibility become clearer, scientists are likely to adapt their methods to make sense of the “multiverse” (Steegen et al., 2016) of analytical approaches available for their computations. Neuroimaging processing and data analysis are undergoing a fundamental change, from a single tool to a multi-tool/multiverse paradigm.

We have discussed solutions pertaining to two important drivers of analytical flexibility, namely the variability resulting from the tools and the computational environments researchers use. Numerous software platforms have surfaced in the neuroimaging community that facilitate investigating the impact of analytical flexibility results, or structure their workflows in reproducible manners where analytical choices are unambiguous by design and can be investigated. This review catalogs in one document recent and open software and platforms, and informs on utility within the context of analytical flexibility.

**Fig. 4.**
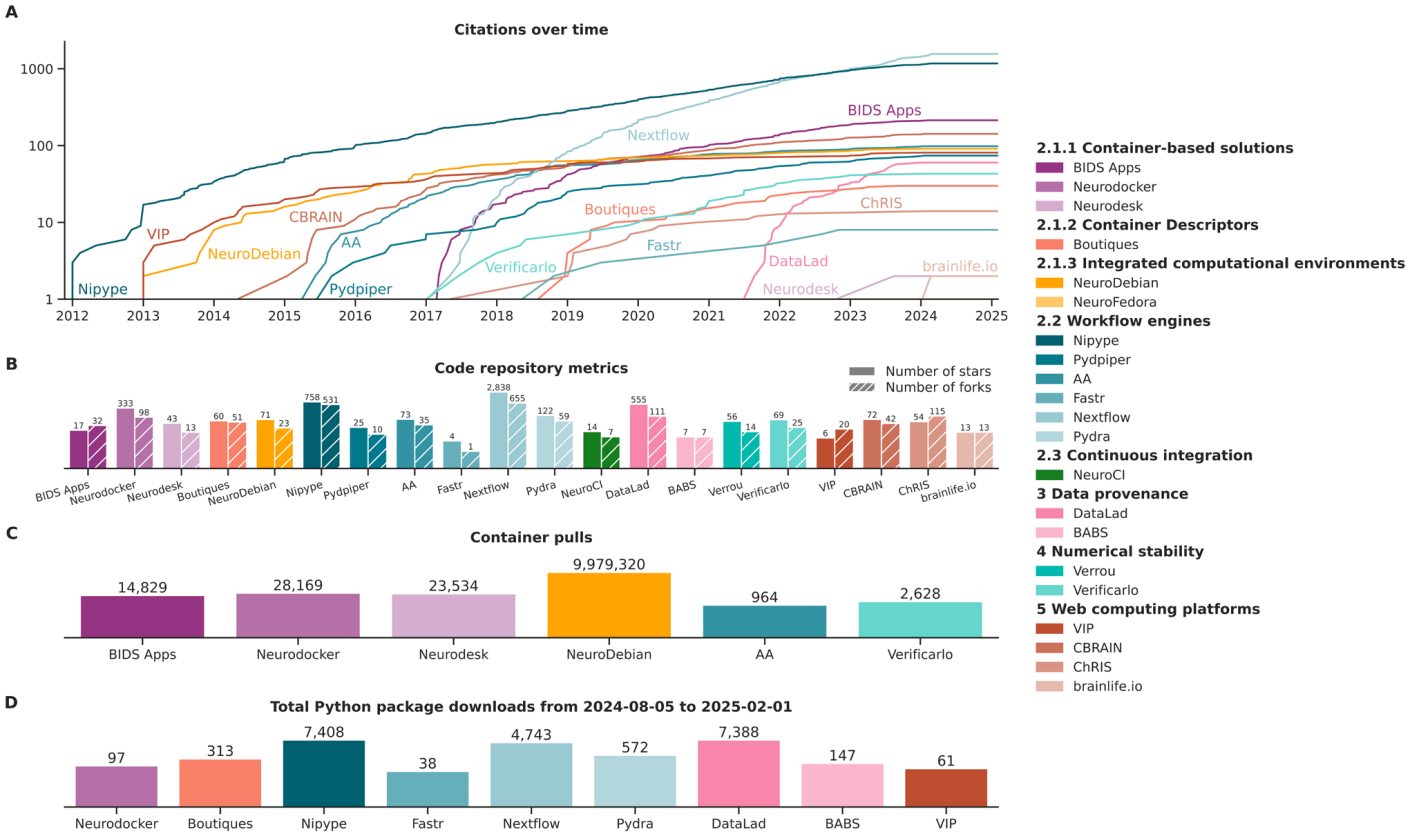
Citation and code metrics for tools described in this review. (A) Cumulative number of citations obtained using OpenCitations (https://opencitations.net/), with some manual corrections for entries with wrong dates. (B) Total number of stars and forks on GitHub (https://github.com/) or GitLab (https://about.gitlab.com/), as obtained through their respective Application Programming Interfaces (APIs). (C) Total number of times container images were downloaded from Docker Hub (https://hub.docker.com/), as obtained from its API. (D) Total number of times Python packages were downloaded from the Python Package Index (PyPI, https://pypi.org/) and/or a Conda channel (https://docs.conda.io/en/latest/) from August 2024 to February 2025, as obtained through the pypistats (https://pypistats.org/) and condastats (https://condastats.readthedocs.io/en/latest/) Python packages, respectively. All code and data used to generate these plots can be found at https://github.com/neurodatascience/analytical-flexibility-tool-metrics. Note that the number of citations is based on information available in OpenCitations and may not be entirely accurate. Moreover, in some cases we used citation metrics from the preprint version of a paper because the final published version was not indexed in OpenCitations.

Figure 4 summarizes adoption and usage trends for the tools discussed, using metrics such as citations, GitHub stars, and download statistics. These trends reveal the growing role of specific tools in reproducible research and provide insights into their community impact and ongoing relevance.

In addressing these challenges, we recommend adopting multiple tools when performing analyses to assess result variability across tools, parameters, and environments. Practical steps for researchers include utilizing container-based solutions or integrated computational environments to simplify the usage of diverse tools and environments. For complex sequences of computations across tools and parameters, using one or a combination of workflow engines, data provenance tools, and container descriptors ensures that analyses can be re-executed unambiguously and shared. For more technically advanced researchers, implementing CI runs to automatically re-execute experiments across evolving tools and data further enhances and automates reproducibility. Software and pipeline developers in particular should consider benchmarking the numerical stability of their products. These recommendations provide clear, actionable thresholds for researchers looking to investigate analytical flexibility, and improve the robustness of their neuroimaging studies. To help users select the most suitable platforms for addressing these scenarios, we have provided Table 3, which outlines the criteria used for evaluation, followed by Table 4, which condenses our evaluation and discussion into a structured comparison based on those criteria. These include Setup Ease, Usage Simplicity, Documentation Quality, Required Technical Expertise, Experiment Feasibility (focused on brain extraction), Local/Remote Execution, and Provenance Information. This table offers a clear, at-a-glance reference to guide researchers in choosing the right tools for their needs.

**Table 3.** Criteria for platform evaluation.

Category	Format	Method
Setup Ease (Acquisition, Installation, Configuration)	Low, Intermediate, High	**Low:** Setup is not documented, dysfunctional, or impossible to download/find. Errors are severe or unsolvable. **Intermediate:** Setup is minimally documented, with solvable errors or challenges. Some steps are unclear but functional. **High:** Setup is well documented, functional, and straightforward. No significant errors encountered.
Usage Simplicity	Low, Intermediate, High	**Low:** Software could not be operated despite significant effort (e.g., 2+ hours of troubleshooting). Critical errors or unclear functionality persist. **Intermediate:** Software works but requires considerable effort to debug, interpret, or operate. Documentation or interface is unclear or only partially helpful. **High:** Software works as documented, with no significant errors. Operation is intuitive, straightforward, and aligns with user expectations.
Documentation Quality	Low, Intermediate, High	**Low:** Documentation is non-existent, severely lacking, or unintelligible. Critical information is missing or errors render it unusable. **Intermediate:** Documentation is minimal but functional, providing basic guidance. Some sections may lack detail or clarity, but the software can still be operated. **High:** Documentation is extensive, functional, understandable, and well organized. It provides clear, detailed, and actionable guidance for tried use-cases.
Required Technical Expertise for End-users	Minimal, Intermediate, or Advanced	**Minimal:** The software is designed for users with minimal technical expertise. It features a GUI/point-and-click interface and requires no knowledge of command–line, coding, or advanced concepts. **Intermediate:** The software requires moderate technical expertise, such as familiarity with the command–line or basic scripting. Minimal coding or file manipulation may be needed, but advanced concepts are not required. **Advanced:** The software demands advanced technical expertise, including knowledge of coding, file manipulation, or application of advanced computing concepts (e.g., server setup, cloud computing, containerization).
Experiment Feasibility	Yes/No.	**Yes:** The tool’s core features for analytical flexibility work as advertised. We were able to carry out the experiment without issues. **No:** The tool’s core features do not work as advertised, or critical functionality is missing or defective, preventing the completion of the experiment.
Local/Remote Execution	Local/Remote, if remote specify which (SGE, SLURM, AWS, etc.)	N.A.
Provenance Information	Yes/No, if yes include the type (Custom, W3C, etc.)	N.A.

**Table 4.** Summary table of the evaluation of the platforms to investigate analytical flexibility in neuroimaging.

Section	Platform Name	Setup Ease	Usage Simplicity	Documentation Quality	Required Technical Expertise for End-users	Experiment Feasibility	Local/Remote Execution	Provenance Information
2.1.1	BIDS Apps	High	High	High	Intermediate	Yes	Both	Tool Dependent
	Neurodocker	High	High	High	Intermediate	Yes	Both	N.A.
	Neurodesk	High	High	High	Beginner	Yes	Both	Tool Dependent
2.1.2	Boutiques	High	High	High	Intermediate	Yes	Both	Custom
2.1.3	NeuroDebian	High	High	High	Intermediate	Yes	Local	No
	NeuroFedora	High	High	High	Intermediate	No	Local	No
2.2	Nipype	High	High	High	Advanced	Yes	Both	W3C PROV
	Pydpiper	Low	N.A.	Low	N.A.	No	Both	Custom
	AA	High	Intermediate	Intermediate	Advanced	Yes	Both	Custom
	Fastr	Intermediate	Low	Intermediate	Advanced	No	Both	W3C PROV
	Nextflow	High	High	High	Advanced	Yes	Both	Custom
	Pydra	High	High	High	Advanced	Yes	Both	Custom
2.3	NeuroCI	Intermediate	Intermediate	Intermediate	Advanced	No	Remote	Custom
3	DataLad	High	High	High	Intermediate	Yes	Both	Git and Custom
	BABS	High	Low	High	Advanced	No	Remote	Git and Custom
4	Verrou	Intermediate	High	High	Advanced	Yes	Both	Floating Point Operation Report
	Verificarlo	Intermediate	Intermediate	Intermediate	Advanced	Yes	Both	No
5	CBRAIN	High	High	High	Beginner	Yes	Remote	Custom
	VIP	High	High	High	Beginner	Yes	Remote	Custom
	brainlife.io	High	High	High	Beginner	Yes	Remote	Custom
	ChRIS	High	High	High	Beginner	Yes	Remote	Custom

While implementing the full array of software packages outlined in this paper may be impractical for many researchers, this work will help researchers strategically select packages to investigate analytical flexibility in their work, thereby bolstering confidence in neuroimaging results. This will impact neuroimaging training and results publishing as the need for platforms enabling analytical flexibility and reporting robustness grows.

Lastly, we would like to acknowledge the community’s efforts in advocating for and developing tools that advance reproducibility in neuroimaging—particularly the ReproNim project, which has partially funded this work and fostered a community from which many such tools have emerged. We also acknowledge the contributions of standards organizations—particularly the International Neuroinformatics Coordinating Facility—whose work underpins the foundations of interoperable computation. Finally, many of these tools, standards, and software are sustained through volunteer-driven and often underappreciated efforts (Westner et al., 2025), and the individuals who ensure their usability deserve our sincere praise and thanks.

## Data Availability

The single-subject test dataset ds005072 from OpenNeuro is available at https://openneuro.org/datasets/ds005072/versions/1.0.1.

## Appendix

**Appendix Table A1.** Summary of the tools discussed in Pipeline Containerization and Tool Repositories (Section 2.1).

Tool	Category	License	Analysis Tool Integration	User Interface	OS compatibility	Unique Features
BIDS Apps	Container-Based Solution	Creative Commons (CC) BY 4.0	Docker containers	Docker or Singularity/Apptainer	Platform Independent	Portable containerized application framework with BIDS data ingestion, and harmonized tool interfaces.
Neurodocker		Apache 2.0	Docker containers	Docker or Singularity/Apptainer	Platform Independent	Easily create custom containers with the specific environment and tool versions you want.
Neurodesk		Berkeley Software Distribution (BSD) 3-Clause	Docker containers	Docker or Singularity/Apptainer, local installation, web-browser/server, or command line	Platform Independent	Modular data analysis environment that leverages an extensive offering of containerized tools, and a wide array of user interfaces catering to many technical skill levels.
Boutiques	Container Descriptor	MIT (Massachusetts Institute of Technology)	Docker/ Singularity /rootfs containers	Command–line or Python	Platform Independent	Facilitates the reproducible creation, management, and sharing of container descriptors across computational environments.
NeuroDebian	Computational Environment	General Public License (GPL)-3+	VM with locally installed tools and preconfigured tool repositories	OS	Platform independent as a VM	OS with pre-installed neuroimaging tools.
NeuroFedora		MIT		OS		

**Appendix Table A2.** Summary of the tools discussed in Workflow Engines (Section 2.2).

Tool	License	Data Input Structure and Format	Tool Integration Mechanisms	User Input Format	Provenance Format	Checkpointing	Unique Features	OS Compatibility	Execution Environment
Nipype	BSD	User specified (via Python string formatting)	Executable tools described in Interface objects.	Python	World Wide Web Consortium Provenance (W3C PROV)	Yes	Facilitates combining processing steps from different tools.	Platform independent	Local, cluster.
Pydpiper	Modified BSD	List of user-specified Medical Imaging NetCDF (MINC) files	Executable tools described or Singularity containers described in Jinja2 syntax templates.	Python	Custom	Yes	Specializes in image registration	Platform independent	Local, cluster.
AA	MIT License	BIDS datasets, or custom Digital Imaging and Communications in Medicine (DICOM)/Neuroimaging Informatics Technology Initiative (NIfTI) structure.	Executable tools described in MATLAB templates.	MATLAB	Custom	Yes	Oriented toward MATLAB users.	Natively UNiplexed Information Computing System (UNIX). Platform independent BIDS app available.	Local, cluster, cloud.
Fastr	Apache 2.0	Dictionary of data source structures and data types defined by users in plugins, and specific inputs files are searched for with regex. Nifti and DICOM are pre-installed.	Executables Tools or Docker containers described in XML/JSON and a Python configuration file.	Command–line	W3C PROV	No	Works with “*virtually any image archive and processing infrastructure*”	Platform independent.	Local, cluster, grid, cloud.
Nextflow	Apache 2.0	Customizable via DSL.	Executables or Singularity/Docker containersdescribed in DSL files.	Command–line and custom DSL configurations based on Groovy.	Custom	Yes	Not specialized towards neuroimaging.	POSIX	Local, cluster, grid, cloud.
Pydra	Apache 2.0	Customizable via Python Classes.	Executables, Python functions, or Singularity/Docker containers are described in a Python Task class.	Python	Custom	Yes	Highly flexible and has advanced features (Nested and Hashed Workflows, map/reduce, mapping splitters, etc.)	Platform independent.	Local, remote via Dask (cluster, grid, cloud).

**Appendix Table A3.** Summary of NeuroCI, discussed in Continuous Integration (Section 2.3).

Tool	Data Input Structure and Format	Tool Integration Mechanisms	User Input Format	Provenance	Workflow Checkpointing	OS Compatibility	Unique Features
NeuroCI	Each dataset’s imaging files in a flat folder structure	Boutiques-described containers on CBRAIN.	YAML experiment definition, JSON configuration files.	Custom JSON database	Yes	Platform independent	Can continuously execute multiple analysis pipelines on multiple datasets, leveraging a compute cluster.

**Appendix Table A4.** Summary of the tools discussed in Data Provenance (Section 3).

Tool	Data Input Structure and Format	Tool Integration Mechanisms	User Input Format	Provenance	License	OS Compatibility	Unique Features
DataLad	Any structure/format	Any shell command (records its impact on dataset)	Command–line, Python library.	Git history log, or “recorded” custom JSON.	MIT	Platform independent.	Allows users to track changes to their datasets over time by adapting the Git workflow for software development
BABS	BIDS	Containerized BIDS apps	Command–line	Git history log, or “recorded” DataLad JSON.	MIT	UNIX HPCs with SLURM or SGE schedulers.	Integrated and reproducible execution and provenance of tools (BIDS Apps) and datasets (DataLad).

**Appendix Table A5.** Summary of the tools discussed in Numerical Stability (Section 4).

Tool	User Input Format	License	OS Compatibility	Unique Features
Verrou	Command–line	GPL 2.0	Linux, FreeBSD, Solaris, Darwin, Android	Language-agnostic MCA analysis tool. Available as an extension/patch for a memory debugging tool suite.
Verificarlo	Command–line	Apache 2.0	Linux. Platform independent via available Docker container	MCA analysis tool for C, C++, and Fortran. Available as an extension for a compiler.

**Appendix Table A6.** Summary of Neuroimaging Web Computing Platforms (Section 5).

Name	License	Tool Integration Mechanisms	Internals	Intended Modalities & Use-Cases	Instances & Deployment
Virtual Imaging Platform (VIP)	CEA CNRS INRIA Logiciel Libre - B (CeCILL-B)	Boutiques-described containers, or executables packaged in Java modules.	Workflows are described using “Grid Workflow Environment Description in Application” (GWENDIA), scheduled using “Distributed Infrastructure with Remote Agent Control” (DIRAC), and executed using the “Modular Optimized Environment for Workflow Execution and Remote computing” (MOTEUR) workflow engine. Data management is based on “Disk Pool Manager - Storage Element” (DPM-SE). The web-portal client-server architecture is based on the Java servlet class.	Aimed for medical image simulation — but generalizes to other domains. Is successfully used in other applications such as cartographies/mappings, segmentations, mean-Shift filtering, neuroimaging, etc.	Main instance hosted on the European Grid Biomed distributed infrastructure.
CBRAIN	GPL 3.0	Boutiques-described containers.	Ruby on Rails for server deployment and web-application (“Bourreau” module for the backend, “Brainportal” for the frontend).	Can use data and tools from any discipline.	Main instance is in the McGill Centre for Integrative Neuroscience.
ChRIS	MIT	Any Linux-executable (compiled C/C++ code, Python scripts, bash scripts, etc.), deployed in a specific Python wrapper.	Linux environment as a base, Apache2 for server deployment, PHP and JavaScript for web development, MySQL database to track data states.	Can use any data type. Primarily aimed at a variety of 2D/3D/4D medical image data formats.	Multiple deployments: Boston Children’s Hospital, Massachusetts General Hospital, Massachusetts Green High Performance Compute Center, Cape Universities Brain Imaging Center. Also available as a virtual machine for users to deploy.
Brainlife.io	MIT	Any executable deployed alongside their specific JSON configuration template.	Uses a large array of JavaScript based tools to build its modules: React and Vue are used for the frontend web interface, Redis for managing connections, influxDB to keep track of resources for management, MongoDB to track real time events, and “Node Package Manager” (NPM) for package management.	Generically multimodal — many different data types are available, and users can register new data types.	The main instance runs out of The University of Texas at Austin, but uses diverse computing centers.
